# Combined structural and functional imaging reveals cortical deactivations in grapheme-color synaesthesia

**DOI:** 10.3389/fpsyg.2013.00755

**Published:** 2013-10-30

**Authors:** Erik O'Hanlon, Fiona N. Newell, Kevin J. Mitchell

**Affiliations:** ^1^School of Psychology and Institute of Neuroscience, Trinity College DublinDublin, Ireland; ^2^Smurfit Institute of Genetics and Institute of Neuroscience, Trinity College DublinDublin, Ireland

**Keywords:** VBM, fMRI, diffusion, DTI, structural, negative BOLD, deactivation, synesthesia

## Abstract

Synaesthesia is a heritable condition in which particular stimuli generate specific and consistent sensory percepts or associations in another modality or processing stream. Functional neuroimaging studies have identified potential correlates of these experiences, including, in some but not all cases, the hyperactivation of visuotemporal areas and of parietal areas thought to be involved in perceptual binding. Structural studies have identified a similarly variable spectrum of differences between synaesthetes and controls. However, it remains unclear the extent to which these neural correlates reflect the synaesthetic experience itself or additional phenotypes associated with the condition. Here, we acquired both structural and functional neuroimaging data comparing thirteen grapheme-color synaesthetes with eleven non-synaesthetes. Using voxel-based morphometry and diffusion tensor imaging, we identify a number of clusters of increased volume of gray matter, of white matter or of increased fractional anisotropy in synaesthetes vs. controls. To assess the possible involvement of these areas in the synaesthetic experience, we used nine areas of increased gray matter volume as regions of interest in an fMRI experiment that characterized the contrast in response to stimuli which induced synaesthesia (i.e., letters) vs. those which did not (non-meaningful symbols). Four of these areas showed sensitivity to this contrast in synaesthetes but not controls. Unexpectedly, in two of them, in left lateral occipital cortex and in postcentral gyrus, the letter stimuli produced a strong negative BOLD signal in synaesthetes. An additional whole-brain fMRI analysis identified 14 areas, three of which were driven mainly by a negative BOLD response to letters in synaesthetes. Our findings suggest that cortical deactivations may be involved in the conscious experience of internally generated synaesthetic percepts.

## Introduction

Synaesthesia is a heritable condition in which particular stimuli generate specific and consistent sensory percepts or associations in another modality or processing stream (Galton, 1883; Cytowic, 1989/2002; Baron-Cohen et al., 1993). Many different forms exist, including colored letters or words (grapheme- or linguistic-color synaesthesia), “colored hearing,” words to taste, tastes to shapes, music to color or shapes, the association of numbers or calendar units with spatial locations and many others (Rich and Mattingley, 2002; Ward, 2013). The condition is quite common, with between 1 and 4% of the population estimated to have the condition (Simner et al., 2006; Simner, 2012).

Though originally defined as a cross-sensory phenomenon, many cases involve cognitive or higher-level conceptual inducers and/or concurrents (Barnett et al., 2008a; Simner, 2012). Synaesthesia may thus, be better conceptualized as the association of additional attributes into the schema of the inducing object (Mitchell, 2011). The synaesthetic experience is characterized by conscious awareness of the concurrent, either as a vivid sensory percept—perceived externally (for “projector” synaesthetes) or “in the mind's eye”—or as an integral attribute brought to mind by the inducing stimulus (in the way that yellow is brought to mind by thinking of a banana) (for “associator” synaesthetes).

The mechanism driving these additional percepts or associations is unknown. In theory, it could involve cross-activation from a cortical area representing the inducing stimulus to one representing the concurrent percept or association. This cross-activation could be mediated by direct connections (Hubbard et al., 2005; Hubbard, 2007a) or indirectly, via an additional area or areas, possibly through feedback connections (Grossenbacher and Lovelace, 2001; Ward and Mattingley, 2006; Neufeld et al., 2012). Alternatively, the synaestheic experience could involve “hyper-binding” between cortical areas (Weiss and Fink, 2009; Van Leeuwen et al., 2010; Rouw et al., 2011), where some mechanism such as the synchronization of cortical oscillations drives the co-activation and thus, the mental association of patterns of activity representing the inducer and concurrent. While such synchronization may indeed be required it seems insufficient to explain the arbitrary, idiosyncratic, and stable nature of the synaesthetic associations—for example, for A to be bound to olive green, the representation of olive green would still have to be activated in the first place. An integrated model proposes that the synaesthetic experience may need both cross-activation and perceptual binding in order to engage frontal areas required for conscious awareness (Dehaene and Changeux, 2011; Hubbard et al., 2011).

A number of functional and structural neuroimaging experiments have been performed to try to define the neural correlates of synaesthetic experiences and to characterize structural differences associated with the condition. Functional magnetic resonance imaging (fMRI) and electroencephalography studies have provided some insights into the neural basis of synaesthesia but their findings are quite variable (Paulesu et al., 1995; Aleman et al., 2001; Nunn et al., 2002; Hubbard et al., 2005; Weiss et al., 2005; Gray et al., 2006; Rich et al., 2006; Sperling et al., 2006; Steven et al., 2006; Beeli et al., 2008; Barnett et al., 2008b; Goller et al., 2009; Brang et al., 2010). Many fMRI studies have found some anomalous activation of visual areas in response to the presentation of the “inducer”—either aurally presented sounds or visually presented achromatic graphemes. However, in addition to this major conclusion, equally remarkable is the variability of findings, even across studies that investigated the same form of (grapheme-color) synaesthesia. Several studies (Nunn et al., 2002; Hubbard et al., 2005; Sperling et al., 2006; Van Leeuwen et al., 2010) have found extra activation in the region of visual area V4, for example—a region involved in color perception (Lueck et al., 1989; Mckeefry and Zeki, 1997)—but others have not observed this and have seen activation or functional connectivity differences in other visual areas (Aleman et al., 2001; Rich et al., 2006) or in other areas, such as parietal cortex (Weiss et al., 2005; Van Leeuwen et al., 2010; Neufeld et al., 2012). Others have observed no additional activation correlating with the synaesthetic experience (Hupe et al., 2012). A positron emission tomography (PET) study also found, in addition to some areas of extra activation in colored-hearing synaesthetes, greater cortical *deactivation* in other areas in response to spoken words which induced a synaesthetic experience of color. These differential effects were induced selectively by words but not tones, in synaesthetes but not controls (Paulesu et al., 1995).

Phenotypic heterogeneity, including between “projector” and “associator” synaesthetes may explain some of the variation in these results (Hubbard et al., 2005; Rouw and Scholte, 2010). Nevertheless, a simple model of excess cross-activation between highly restricted cortical areas seems too minimal to accommodate all these findings. Rather, these findings suggest that differences in connectivity—either due to structural or functional changes—may be quite extensive in the brains of synaesthetes, a hypothesis which is supported by structural neuroimaging studies.

Several studies have identified structural differences in the brains of synaesthetes compared to controls (Rouw and Scholte, 2007, 2010; Hanggi et al., 2008; Jancke et al., 2009; Weiss and Fink, 2009; Banissy et al., 2012b). In almost all cases, synaesthetes showed greater volumes of areas of gray or white matter or greater fractional anisotropy (FA) within certain white matter tracts than controls [see Banissy et al. (2012b) for an exception]. Some of these differences are in the general region of visual areas thought to be involved in the synaesthetic experience but others are more widespread, in parietal or even frontal regions. A recent study analyzed global connectivity patterns in the brains of synaesthetes, using networks derived from correlations in cortical thickness (Hanggi et al., 2011). The global network topology was significantly different between synaesthetes and controls, with synaesthetes showing increased clustering, suggesting global hyperconnectivity. The differences driving these effects were widespread and not confined to areas hypothesized to be involved in the grapheme-color synaesthetic experience itself. Widespread functional connectivity differences have also been observed in a study using resting-state fMRI (Dovern et al., 2012).

Given the variability of functional imaging findings in synaesthesia, in particular the inconsistency of activation of specific visual areas such as V4, or indeed of any visual areas, we adopted an unbiased approach to look for differences in functional responses in synaesthetes vs. controls. We first carried out a whole-brain volumetric and diffusion tensor imaging (DTI) analysis to identify regions of structural differences between groups of synaesthetes and controls. We then used the clusters of gray matter difference as regions of interest for functional analyses, identifying differential sensitivity to the contrast between letters and non-meaningful characters in synaesthetes compared to controls. In parallel, we conducted a whole-brain functional analysis based on responses to visual stimuli that do or do not induce synaesthetic percepts between synaesthetes and non-synaesthete controls. Surprisingly, several of the functional differences we observe are driven by negative blood oxygen level-dependent (BOLD) response, reflecting unexpected cortical deactivations in this sample of synaesthetes in response to letters.

## Methods

### Participants

We recruited 24 right handed participants for our study through local and media advertising, and from the student population at Trinity College Dublin. All participants were female, with a mean age of 38.76 years, and an age range of between 20–58 years. This sample included 13 synaesthetes and 11 non-synaesthete controls and both groups were matched for age (mean ages of 38.1 years, *SE* = 3.6 and 37.1 years, *SE* = 4.2, for the synaesthete and non-synaesthete groups, respectively). Synaesthetes were identified by repeated testing for consistency of their letter-color associations over time. The details of the consistency tests used are described in Barnett et al. (2008a). None of our participants reported a history of neurological disorders or psychiatric diagnoses, substance abuse, or were treated at any time with psychiatric medications. All had normal or corrected-to-normal vision, and none reported any auditory deficits. The experimental protocol was approved by the School of Psychology Ethics Committee, Trinity College Dublin and all participants gave written informed consent to participate prior to the study.

### MRI scanning parameters

#### Anatomical scanning protocol

All scanning was conducted on a Philips Achieva 3.0 T scanner, fitted with an eight channel head coil, equipped with a mirror that reflected the display projected on a 640 × 480 panel. This panel was placed behind the participant's head, outside the magnet. The mirror was mounted on the head coil in the participant's line of vision. 180 axial high-resolution T1-weighted anatomical Spoiled Gradient Echo (SPGR) images [Echo Time (*TE*) = 3.8 ms, Relaxation Time (*TR*) = 8.4 ms, Field Of View (FOV) = 230 mm, 162 mm, 0.898 × 0.898 mm^2^ in-plane resolution, slice thickness 0.9 mm, flip angle α = 8°] were acquired before the first functional imaging, to allow for subsequent activation localization and spatial normalization of fMRI data and for the purpose of Voxel Based Morphometry (VBM) analyses.

#### DTI scanning protocol

Diffusion weighted images were obtained using spin-echo, echo-planar imaging (SE EPI) pulse sequence (*TE* = 52 ms, *TR* = 11,260 ms, flip angle α = 90°, FOV = 224 mm, 149.7 mm). 60 axial slices were acquired with an in-plane resolution of 1.75 × 1.75 mm^2^, a slice thickness 2.5 mm, and a gap = 0.3 mm. Data with a b-value = 800 s mm^−2^ and 15 non-collinear gradient directions was collected. The start of each series of directions was preceded by the acquisition of a non-diffusion-weighted volume (*b* = 0) for the purpose of image registration and motion correction.

#### fMRI scanning protocol

The task was preceded by approximately 20 min of standard scout images, (including shimming to reduce the EPI image artifacts), and SPGR structural acquisitions. Thirty-two, non-contiguous (10% gap) 3.5 mm axial slices covering the entire brain were collected using a T2* weighted echo-planar imaging sequence (*TE* = 35 ms, *TR* = 2000 ms, flip angle α = 90°, FOV = 224 mm, 122.85 mm, 64 × 64 matrix size, 1.75 × 1.75 mm^2^ in-plane resolution). Imaging used a parallel SENSitivity Encoding (SENSE) approach with a reduction factor = 2.

### MRI data analysis protocols

#### Image pre-processing

The MR images were collected in Philips PAR and REC format and were converted to NIFTI file format for the purposes of VBM and diffusion tensor image analyses including tractography. Data acquired for the purpose of functional MRI analysis was converted to AFNI HEAD and BRIK file format and analyzed using the AFNI software tools (http://afni.nimh.nih.gov).

#### Whole-brain structural analysis using FSL-VBM

To investigate possible structural brain differences between synaesthetes and non-synaesthetes a voxel-wise whole brain “optimized” VBM-style analysis was performed using FMRIB FSL VBM tools (Smith et al., 2004). VBM is a voxel-wise automated analysis technique performed on high resolution structural images to investigate differences in local concentrations or volumes (with the inclusion of a modulation step) of gray and white matter [see Ashburner and Friston (2000); Good et al. (2001) for detailed descriptions of the standard and optimized VBM methods]. Briefly, structural images were brain-extracted using BET (Smith, 2002) and segmented before being registered to the MNI 152 standard space using non-linear registration (Andersson et al., 2007b). The resulting images were averaged and flipped along the x-axis to create a left-right symmetric, study-specific gray matter, white matter, and cerebrospinal fluid templates. Second, all native gray and white matter images were non-linearly registered to these study-specific templates and “modulated” to correct for local expansion (or contraction) due to the non-linear component of the spatial transformation. The modulated gray and white matter images were then smoothed with an isotropic Gaussian kernel with a sigma of 2.5 mm (5.75 FWHM).

#### VBM statistical analysis

MRICron was used for the purpose of voxelwise non-parametric statistical tests featuring Brunner Munzel tests for the purpose of group comparisons and correction for multiple comparisons using False Discovery Rate (FDR). These were conducted for the purpose of group comparisons and the identification of global gray and white matter volume differences. Significant voxels passed a voxelwise statistical FDR threshold of *p* = 0.01 corrected and a minimum cluster size threshhold of 10 mm^3^.

#### Whole-brain DTI white matter analysis using FSL

Data analysis was performed with FSL. Pre-processing featured eddy current and movement correction. Voxelwise statistical analysis of the FA data was carried out using (TBSS) Tract-Based Spatial Statistics, (Smith et al., 2006), which is part of FSL (Smith et al., 2004). First, FA images were created by fitting a tensor model to the raw diffusion data using FDT, and then brain-extracted using BET (Smith, 2002). All participants' FA data were then aligned into a common space using the non-linear registration tool FNIRT (Andersson et al., 2007a,b), which uses a b-spline representation of the registration warp field (Rueckert et al., 1999). Next, the mean FA image was created and thinned to create a mean FA skeleton, which represents the centers of all tracts common to the group. Each participant's aligned FA data was then projected onto this skeleton and the resulting data fed into voxelwise, cross-subject statistics.

#### DTI statistical analysis

MRICron was used for the purpose of voxelwise non-parametric statistical tests featuring Brunner Munzel tests for the purpose of group comparisons and correction for multiple comparisons using FDR. Significant voxels passed a voxelwise statistical threshold of *p* ≤ 0.01 with a cluster size criterion based on the skeletonized images of 5 mm^3^.

#### Structure/function region of interest (ROI) anlayses (GM VBM-derived).

The gray matter VBM results, (ROIs), showing significant volume differences between synaesthetes and non synaesthete controls were used as mask regions and applied to the functional MRI activation measures for each of the four independent fMRI stimulus conditions (color, achromatic, letter and non meaningful character). Separate 2 × 2 mixed ANOVAs were conducted based on responses to each of the stimulus conditions (either letters vs. non-letters or colors vs. achromatic) with group (synaesthetes or controls) as a between-subjects factor and each of the stimulus conditions as within-subjects factors to examine a main effect for group, for condition and possible group × stimulus condition interactions. *Post-hoc* analyses were carried out to determine the directions of any effects using IBM SPSS version 19 statistical software.

### Experimental design for fMRI test

**Figure 1:**
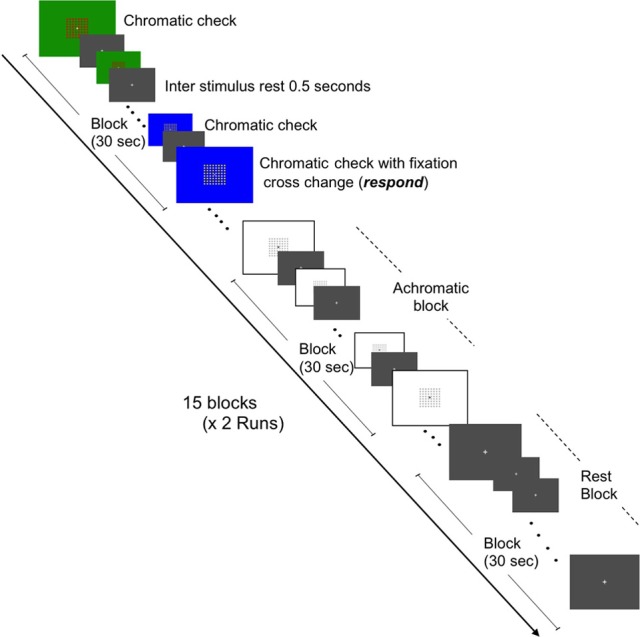
**Schematic representation of fMRI task for brain activation in response to chromatic check patterns and color localization.** Representations of the three conditions (chromatic, achromatic, and rest states) are depicted. Two runs (7 min and 30 s each) containing 15 blocks (30 s in duration) and consisting of equal amounts of each condition, i.e., five blocks of each condition per run were constructed.

**Figure 2:**
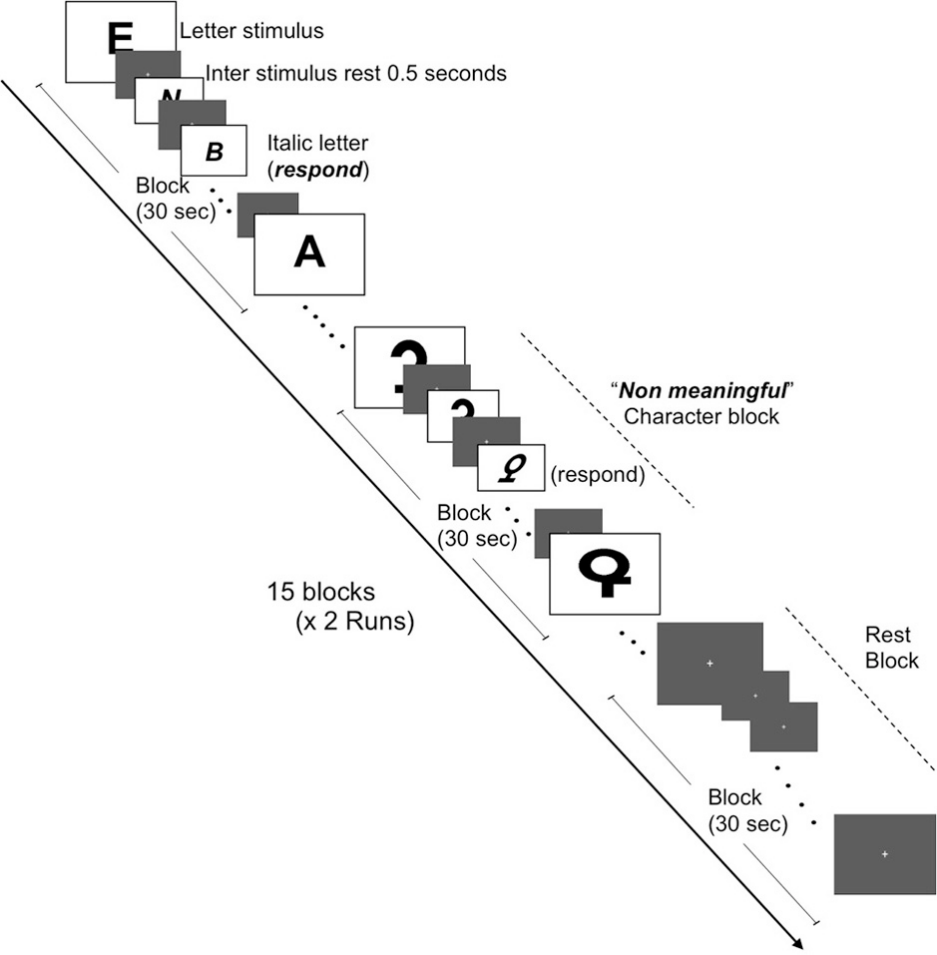
**Schematic of fMRI task for brain activation in response to meaningful letters and non-meaningful characters.** Representations of the three conditions (letters, non-meaningful characters, and rest states) are depicted. Two runs (7 min and 30 s each) containing 15 blocks (30 s in duration) and consisting of equal amounts of each condition, i.e., five blocks of each condition per run were constructed.

Two independent functional MRI tasks were employed to assess the BOLD response to chromatic or achromatic check patterns and graphemes which were meaningful or “non-meaningful” letters. Our grapheme stimulus set were adopted from those previously described by Pesenti et al. (2000). Stimulus presentations were projected onto a viewing screen behind the magnet bore and viewed by the participants via a mirror attached to the head coil, The fMRI sessions consisted of two conditions, namely the Color Images session and the Grapheme Images session. Each session featured two runs of 15 blocks (each block lasting 30 s) with each run containing equal quantities of three conditions, namely rest, chromatic check patterns and achromatic check patterns and rest, letters and non-meaningful characters, respectively. The presentation order of the condition blocks was pseudo randomized across participants. The images were presented at a rate of 0.5 Hz and alternated with a gray background image to reduce after-image effects (Figures 1, 2). Each gray background image for both inter-stimulus and rest condition blocks featured a fixation cross at the center, upon which the participant was instructed to gaze.

#### Color images session

BOLD activations were acquired whilst the participant viewed chromatic and achromatic check pattern stimuli. The chromatic blocks consisted of images of check patterns, viewed in either red/green or blue/yellow combinations. Each check pattern featured a fixation cross at the center, upon which the participant was instructed to gaze. In order to ensure the participant viewed the center of the images, they were instructed to respond whenever a change occurred to the shape of the fixation cross (i.e., from the normal cross to a star shape). Participants responded via a button-press response mechanism was utilized to avoid head motion associated with verbal responses. The change in fixation cross occurred at a rate of four times per block in a pseudo random order used to avoid predictability.

#### Grapheme images session

Changes in BOLD activations were acquired whilst the participants viewed images of graphemes (meaningful letters and non-meaningful characters which shared many of the same visual features as the meaningful letters). As before in the color stimulus presentations, each gray background image for both inter-stimulus and rest condition blocks featured a fixation cross at the center, upon which the participant was instructed to gaze. Participants were required to respond when the letters or non-meaningful characters appeared in an “italic” font format. The change in letter font occurred at a rate of four times per block in a pseudo random presentation to avoid predictability.

### fMRI data analysis

The fMRI data were analyzed using the AFNI software tools (http://afni.nimh.nih.gov) (Cox, 1996). Following image reconstruction, both runs for each session, (i.e., color and grapheme stimuli) were concatenated and motion corrected using 3-D volume registration (least-squares alignment of three translational and three rotational parameters). Noise related artifacts outside the brain were also removed using edge detection techniques.

A block analysis was performed to estimate the activation for the chromatic and achromatic conditions (for the color task) and for the letters and non-meaningful characters conditions (for the grapheme task) separately. The on–off block regressors were convolved with a standard haemodynamic response to accommodate the lag time of the BOLD response. Multiple regression analyses were then used to determine the average level of BOLD activation relative to the rest state periods (baseline). The baseline activations were also derived by averaging the rest periods in each block over both runs for each separate task.

The percentage change map (block activation) voxels were resampled to 1 × 1 × 1 mm voxel resolution, aligned to their T1 anatomy image and then warped into Talairach standard space and spatially blurred with a 3-mm isotropic rms Gaussian kernel.

#### fMRI “whole-brain” voxelwise analysis

Separate whole-brain voxelwise analyses, based on each of the stimulus conditions (i.e., color/achromatic and letter/character contrasts) were conducted based on a Two-Way mixed ANOVA with group as the between-subject factor and stimulus condition as the within-subject factor. Each 2 × 2 ANOVA was used to assess for main effects of group (synaesthete or controls), or stimulus condition (color/achromatic or letters/characters) and for any Group × Condition interactions. Significant voxels passed a voxelwise statistical threshold (*t* = 17.19, *p* < 0.0005, *N* = 22) and were required to be part of a minimum cluster of 134 mm^3^ of contintiguous significant voxels. Thresholding was determined through 5000 Monte Carlo simulations and resulted in a 1% probability of a cluster surviving due to chance, fully corrected for multiple corrections. Resultant thresholded cluster maps were examined through *post-hoc* statistical analysis using a cluster based ROI approach to determine the effect of each condition per significant region of interest. This entailed extracting the mean activation levels for each task condition per cluster and conducting between group *t*-tests per condition. These values were then extracted to SPSS for the purposes of cluster-level statistical analysis and featured Bonferroni correction at *P* = 0.05. In the case of the color/achromatic contrast, the thresholded maps were inspected to ensure activation of color selective regions in response to task stimuli at a threshold of *p* = 0.05 corrected using the same method described above (*t* = 18.68, *p* < 0.005, *N* = 22).

## Results

### Whole brain analyses

#### Gray matter volume comparison

**Figure 3:**
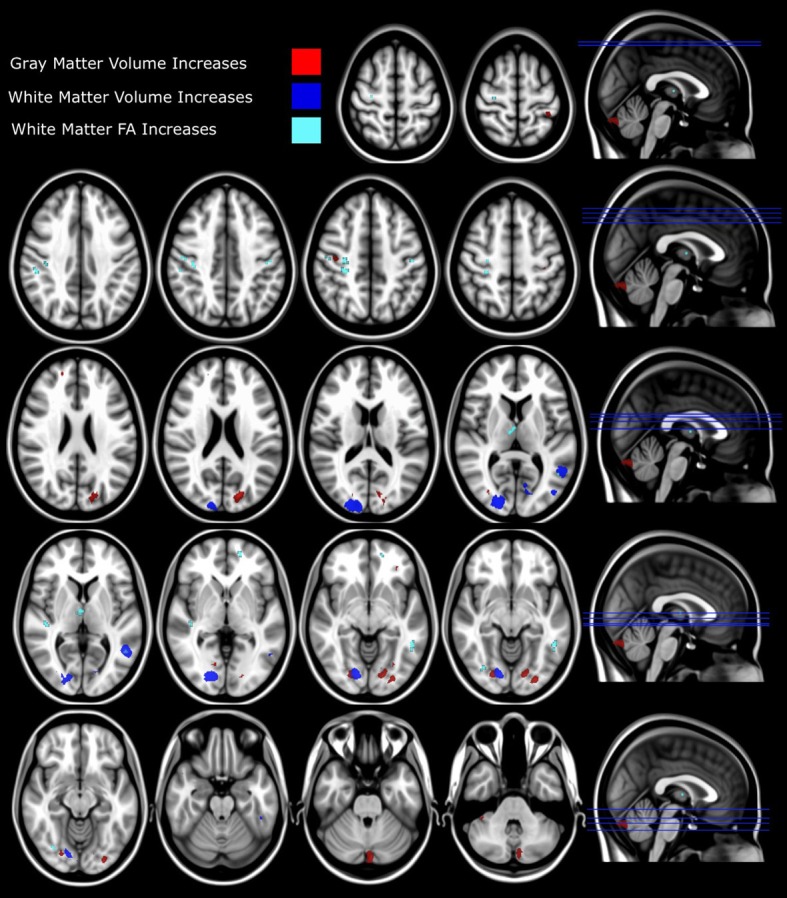
**Structural differences between synaesthetes and non-synaesthete controls.** Whole brain VBM analysis results are shown with significant between-group differences (*p* = 0.01 FDR corrected) for gray matter shown in red (refer to Table 1) and white matter (*p* = 0.01 FDR corrected) shown in blue (refer to Table 2). The slice location of each volumetric difference is indicated on the right hand side of the image. In addition, significant between-group differences from the whole-brain DTI TBSS FA analysis (*p* = 0.05 FDR corrected) are also shown in cyan (refer to Table 3). For all measures (GM, WM, and FA), the effect direction for the significant between-group differences identified was: synaesthetes > non-synaesthete controls. All significant clusters of between-group differences are overlaid on the standard MNI 152 T1 image provided within the FSL toolbox.

Gray matter volumetric analysis results are presented in Table 1. The non-parametric FDR *t*-test (*p* ≤ 0.01 and a minimum cluster size criteria of 10 mm^3^) revealed nine regions of increased gray matter volume in synaesthetes relative to controls (see Figure 3 and Table 1). These regions included bilateral cerebellum, left lateral occipital cortex including the precuneus, the left lateral occipital cortex/fusiform gyrus, bilateral occipital cortex/fusiform gyrus, right lingual gyrus, the right posterior division of the post-central gyrus and the right post-central/pre-central gyrus. No clusters showed a greater volume in controls.

**Table 1:** **Gray matter volume difference (whole brain VBM analysis)**.

**Structure/location**	**Right/left**	**Volume (mm^3^)**	**MNI Coordinates**			***Z*-stat**	**Effect direction**
			***x***	***y***	***z***		
Cerebellum	L	1104	−2	−82	−30	5.17	Syn > Ctrl
Cerebellum/culmen	R	88	10	−40	−34	4.00	Syn > Ctrl
Lateral occipital cortex/precuneus	L	880	−14	−82	20	5.75	Syn > Ctrl
Lateral occipital/occipital fusiform gyrus	L	432	−28	−86	−8	5.06	Syn > Ctrl
Occipital fusiform/lingual gyrus	L	616	−14	−84	−4	5.20	Syn > Ctrl
Occipital fusiform/lingual gyrus	R	456	20	−78	−10	4.14	Syn > Ctrl
Lingual gyrus	R	152	14	−68	0	4.96	Syn > Ctrl
Post−central gyrus, superior division	L	144	−38	−38	60	4.14	Syn > Ctrl
Post−central/pre−central gyrus	R	138	42	−18	48	3.91	Syn > Ctrl

P = 0.01 FDR corrected for multiple comparisons with minimum cluster size = 10 mm^3^. The XYZ coordinates relate to the approximate Center Of Mass (COM) in each case.

#### White matter volume comparison

White matter volumetric analysis results are presented in Table 2. FDR statistical comparisons (*p* = 0.01 with a minimum cluster criteria of 10 mm^3^) revealed six regions of white matter volume increases in synaesthetes compared to controls (see Figure 3 and Table 2). Similar to the gray matter differences, the white matter volume differences again showed increased volumes in the synaesthetes relative to controls and featured the right occiptal pole including the precuneus, left middle occiptal gyrus, left temporal gyrus, left temporal/fusiform gyrus and bilateral lingual gyrus. No clusters showed a greater volume in controls.

**Table 2:** **White matter volume difference (whole brain VBM analysis)**.

**Structure/location**	**Right/left**	**Volume (mm^3^)**	**MNI Coordinates**			***Z*-stat**	**Effect direction**
			***X***	***Y***	***Z***		
Occipital pole/cuneus	R	5776	22	−92	12	7.86	Syn > Ctrl
Middle occipital gyrus	L	88	−44	−80	8	5.39	Syn > Ctrl
Middle temporal gyrus	L	1152	−50	−54	4	6.24	Syn > Ctrl
Temporal/fusiform gyrus	L	80	−48	−38	−22	4.46	Syn > Ctrl
Lingual gyrus	L	312	−14	−78	6	4.22	Syn > Ctrl
Lingual gyrus	R	88	16	−80	0	3.82	Syn > Ctrl

P = 0.01 FDR corrected for multiple comparisons with minimum cluster size = 10 mm^3^. The XYZ coordinates relate to the approximate Center Of Mass (COM) in each case.

#### White matter FA comparison

Whole brain white matter FA was examined and 14 clusters showed significantly increased FA in synaesthetes compared to controls (presented with the GM and WM clusters in Figure 3 and by themselves on a skeletonized framework of axonal tracts in Supplemental Figure 1. See also Table 3). Three regions of increased FA were identified along the right superior longitudinal fasiculus and included parietal/subgyral and supramarginal areas. Two clusters of increased FA were identified along the right inferior longitudinal fasiculus featuring the insula and fronto-occipital and lingual gyrus. The right thalamus showed two clusters of increased FA along the anterior thalamic radiation. There were no clusters showing increased FA in controls compared to synaesthetes.

**Table 3:** **White matter fractional anisotropy FA (whole brain analysis)**.

**Structure/location/tract**	**Right/left**	**Volume (mm^3^)**	**MNI coordinates**			***Z*-stat**	**Effect direction**
			***X***	***Y***	***Z***		
Superior longitudinal fasciculus/ temporal part	L	14	−51	−49	−6	5.14	Syn > Ctrl
Occipital/lingual gyrus (inferior longitudinal fasciculus)	R	9	33	−75	10	5.40	Syn > Ctrl
Temporal cerebral white matter/insula (inferior longitudinal fasciculus)	R	6	45	−23	3	4.16	Syn > Ctrl
Parietal/post−central gyrus	R	8	49	−17	43	4.72	Syn > Ctrl
Parietal/post−central gyrus (superior longitudinal fasciculus)	L	8	−47	−21	44	5.26	Syn > Ctrl
Parietal lobe/subgyral (superior longitudinal fasciculus)	R	7	28	−31	48	4.10	Syn > Ctrl
Subgyral (superior longitudinal fasciculus)	R	6	37	−24	40	5.80	Syn > Ctrl
Supra marginal white matter (superior longitudinal fasciculus)	R	5	50	−30	39	4.37	Syn > Ctrl
Pre−central gyrus (corticospinal tract)	R	6	28	−19	57	4.68	Syn > Ctrl
Frontal gyrus/subgyral	R	6	29	−20	48	5.17	Syn > Ctrl
Frontal cerebral white matter (forceps minor)	L	6	−15	54	−3	5.12	Syn > Ctrl
Thalamus (anterior thalamic radiation)	R	6	6	−11	8	4.23	Syn > Ctrl
Thalamus (anterior thalamic radiation)	R	6	4	−10	6	4.73	Syn > Ctrl
Middle temporal gyrus	L	6	−50	−57	9	4.59	Syn > Ctrl

P = 0.01 corrected for multiple comparisons using FDR. A minimum cluster size criteria of 5 mm^3^ was applied to the fully corrected maps. The XYZ coordinates relate to the approximate Center Of Mass (COM) in each case.

#### fMRI region of interest analysis featuring GM VBM clusters

**Figure 4:**
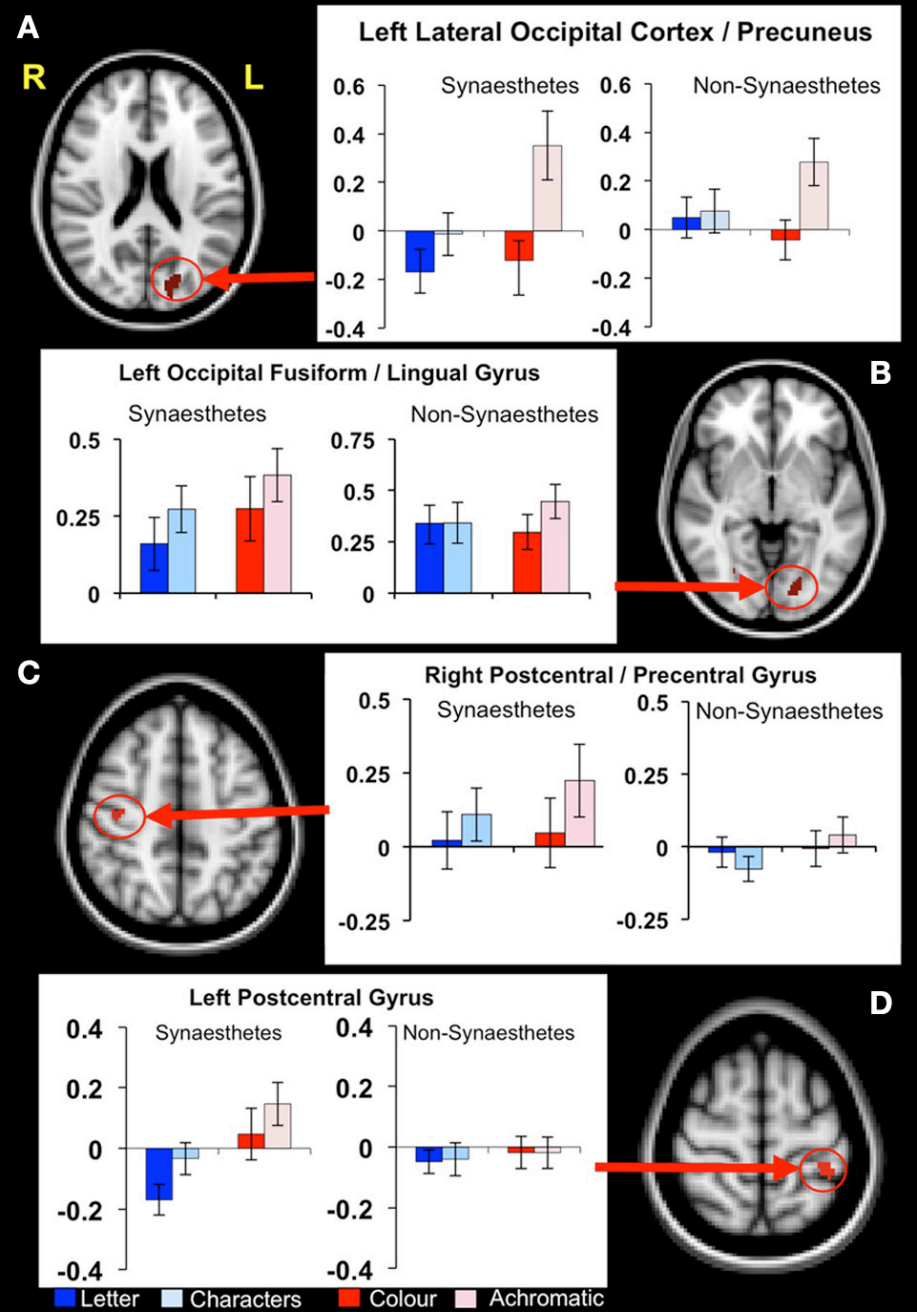
**Functional differences in structurally defined regions of interest.** Four clusters of significantly increased GM volume in synaestehetes showed a significant group × condition interaction for the letter/non-meaningful characters contrast. Mean fMRI task activations within these ROIs are detailed for each task condition (letter/character and color/achromatic) in synaesthetes and controls. Two clusters, in left lateral occipital cortex/precuneus panel **(A)** and in left post-central gyrus panel **(D)** show a main group effect in response to letters (blue bars). These differences are driven by a strong negative BOLD response to letters in synaesthetes. The other two clusters, in left occipital fusiform/lingual gyrus **(B)** and right postcentral/precentral gyrus **(C)** did not show a main group effect for letters.

Using the nine GM VBM clusters, the mean activation levels for each of the fMRI conditions were extracted for the purpose of an ROI analysis to investigate the functional response to the letter/non-meaningful character stimuli at each of these cluster locations. The values were entered into a 2 × 2 ANOVA in SPSS [with group as the between-group measure and condition (letter, non-meaningful characters) as the within-group measure]. The ANOVA did not identify any regions with a significant main effect for either group or condition. Four of the nine clusters showed a significant group × condition interaction for the letter/non-meaningful characters contrast (see Table 4 and Figure 4). Bonferroni correction for multiple testing divides the *p*-value significance cut-off (<0.05) by the number of tests (9 in this case), to generate a corrected significance value for any single test of 0.0056. None of the single clusters pass this corrected significance threshold. However, this only corrects for any one test showing a significant result at *p* < 0.05. The binomial distribution can be used to calculate the likelihood of seeing × out of nine tests being significant at *p* < 0.05, by chance. While the chance of seeing at least 1/9 tests significant at this level is quite high (0.37), the chance of seeing 4/9 tests significant by chance is very low (0.0006). The overall pattern of effects observed is thus highly significant.

**Table 4:** **ROI analysis for fMRI conditions using gray matter VBM defined clusters**.

**Structure/location**	**Mean fMRI activations**			***Post hoc* statistic**	
	**Main effect**		**Interaction**	**(Parameter estimates)**	
	**Group**	**Condition**	**Group × condition**	**Letters**	**Non letters**
Cerebellum	0.519	0.399	0.088	0.182	0.873
Cerebellum/culmen	0.829	0.183	0.506	0.676	0.975
Lateral occipital cortex/precuneus	0.078	0.159	**0.018**	**0.022**	0.267
Lateral occipital/occipital fusiform gyrus	0.938	0.758	0.328	0.722	0.816
Occipital fusiform/lingual gyrus	0.282	0.241	**0.034**	0.141	0.535
Occipital fusiform/lingual gyrus	0.203	0.453	0.388	0.184	0.262
Lingual gyrus	0.553	0.144	0.091	0.344	0.859
Post-central/pre-central gyrus	0.342	0.575	**0.031**	0.860	0.098
Post-central gyrus	0.219	0.499	**0.034**	**0.027**	0.911

ROIs determined by independent VBM analysis. Cluster-based ANCOVA (age corrected) based on mean activations per cluster. Bold text indicated significant cluster. See text for discussion of significance based on multiple tests.

*Post hoc* statistical analyses were used to investigate condition-specific group comparisons. *Post hoc* parameter estimates (letter and non meaningful character conditions) identified two clusters where a greater response for the “letter” condition was found in the synaesthete group, namely the left lateral occipital cortex/precuneus and the right post-central/pre-central gyrus. In both clusters the mean activation levels revealed significantly greater negative BOLD response to letters in synaesthetes compared to controls (Figure 4).

#### fMRI “whole-brain” voxelwise analyses

**Figure 5:**
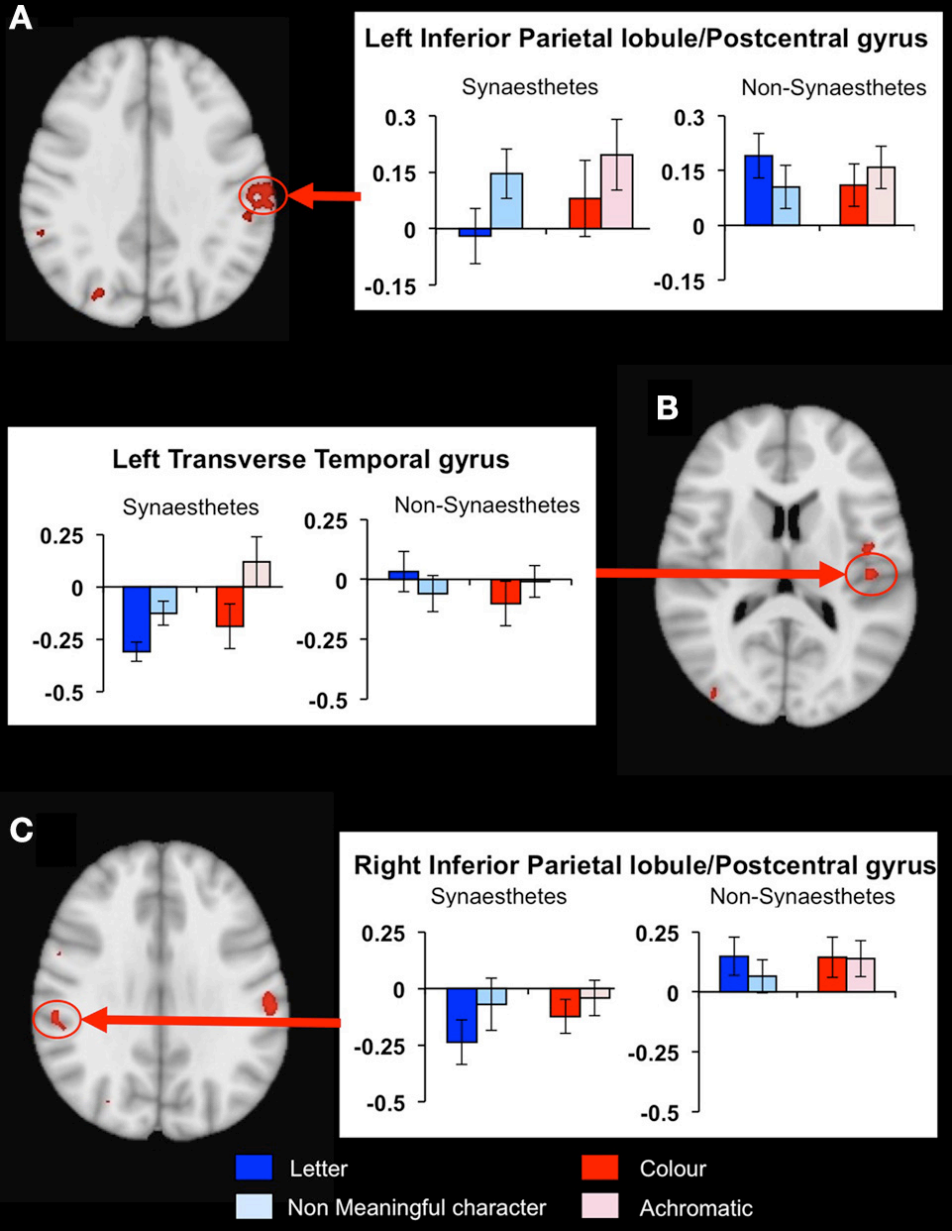
**Results from the whole-brain voxelwise ANOVA for the letter/non-meaningful character contrast.** Three clusters showed a significant group x condition interaction and a main group effect in response to letters. Mean fMRI task activations within these clusters are detailed for each task condition (letter/character and color/achromatic) in synaesthetes and controls. In each case, synaesthetes show a mean negative BOLD response to letters. **(A)** Left inferior parietal lobule/post-central gyrus; **(B)** left transverse temporal gyrus; **(C)** right inferior parietal lobule/post-central gyrus.

To determine whether any other brain areas showed a similar response to letters, or, alternatively, showed the more expected positive BOLD increase, we performed voxelwise whole-brain analyses, looking again for regions showing differential responsiveness to letters vs. non-meaningful characters, in synaesthetes compared to controls. A mixed, 2 × 2 ANOVA using matched samples revealed 14 significant areas showing a group × condition interaction, fully corrected for multiple corrections at a threshold of *P* = 0.01 as detailed in the methods above. *Post hoc* cluster based statistical analyses were carried out to determine the direction of responses driving these group × condition interaction effects. None of these areas showed increased responsiveness to letters vs. characters in synaesthetes but not controls. Three showed a main effect group difference in the response to letters, with synaesthetes having a lower and negative average BOLD response in all three (Table 5). These clusters are in the left and right inferior parietal lobules and the left transverse temporal gyrus (Figure 5). These regions did not overlap the previously defined clusters of increased gray matter volume.

**Table 5:** **Voxelwise ANOVA—Group × Condition Interaction (letters/characters)**.

**Cluster ID**	**Volume (mm3)**	**Location (approximate center of mass)**	**Coordinates(COM)**			**Letters**	**Characters**
			***X***	***Y***	***Z***	***t*-test**	
1	2349	Left inferior parietal lobule/post−central gyrus(BA2/40)	−57	−27	32	**0.019**	0.762
2	546	Left declive (cerebellum)	−43	−63	−20	0.190	0.431
3	505	Left insula (BA13)	−43	−7	6	0.236	0.212
4	246	Right inferior parietal lobule(BA40)	56	−28	24	**0.002**	0.166
5	245	Right supramarginal/inferior parietal lobe (BA40)	57	−40	33	0.104	0.522
6	202	Left medial frontal gyrus(BA6)	−3	−12	54	0.541	**0.047**
7	195	Right cuneus (BA19)	25	−73	31	0.428	**0.004**
8	181	Right superior frontal gyrus(BA6)	25	−2	66	0.705	0.077
9	180	Left precuneus (BA7)	−7	−59	45	0.553	**0.004**
10	166	Right middle occipital gyrus (BA19)	39	−82	13	0.163	0.343
11	158	Left insula/transverse temporal gyrus (BA41/13)	−45	−19	11	**0.002**	0.483
12	149	Right cerebellar tonsil	8	−51	−33	0.940	**0.007**
13	148	Right middle frontal gyrus (BA6)	7	−11	71	0.067	0.362
14	134	Left post−central/ inferior parietal gyrus (BA2)	−45	−27	43	0.075	0.061

Clusters reported were determined through whole brain voxelwise analysis and threshold at p = 0.01 and fully corrected for multiple comparisons. The post hoc between-group t-tests were performed at a cluster-based level and significant values (p < 0.05) are shown in bold.

## Discussion

Several studies have now looked for structural differences in the brains of synaesthetes, using various modalities, with results that show general consistencies but that vary considerably in the details (Rouw and Scholte, 2007, 2010; Hanggi et al., 2008, 2011; Jancke et al., 2009; Weiss and Fink, 2009; Hupe et al., 2012; Zamm et al., 2013). One general point of agreement is that almost all the structural differences observed between synaesthetes and controls, across all studies—whether in cortical thickness, surface area, volume of gray or white matter clusters or FA of white matter—are increases in synaesthetes (Rouw et al., 2011; Hupe et al., 2012; Zamm et al., 2013); [see Banissy et al. (2012b) for an exception, where decreases as well as increases were observed]. This is also true in our study and argues strongly for the validity and generality of these findings on the basis that random differences would be expected to be observed in both directions.

The other trend that is evident across these studies is that though the observed structural differences are concentrated in occipital regions, they are also observed in temporal, parietal, and frontal areas and have even been reported in hippocampus, cerebellum, and thalamus. We see a similar distribution with structural volumetric differences observed most prominently in occipital and temporal areas (including cuneus, lateral occipital cortex, fusiform and lingual gyri), as well as post-central/pre-central gyrus and cerebellum and FA differences apparent in occipital, but also parietal areas and even in thalamic radiations. Though the overall pattern is fairly consistent, no particular locations emerge as a general finding across all these studies.

While the details may vary, the primary picture is quite consistent: synaesthetes strongly tend to show greater gray and white matter volume and greater FA in many regions of the brain. We and others have previously argued that these data provide evidence for a structural difference as the primary cause of synaesthesia (Hubbard, 2007b; Rouw and Scholte, 2007; Bargary and Mitchell, 2008). While these findings are clearly consistent with that model, it has been rightly pointed out that structural differences could of course alternatively arise secondarily due to altered activity patterns in particular brain regions and circuits (Cohen Kadosh and Walsh, 2008). The structural findings thus do not yet lay to rest the question of whether the primary alteration in synaesthesia is anatomical or neurochemical (Grossenbacher and Lovelace, 2001; Ramachandran and Hubbard, 2001; Ward et al., 2006; Hubbard, 2007a; Bargary and Mitchell, 2008).

The fact that the structural differences are quite widespread is consistent with the view that the synaesthetic experience may be just one manifestation of a wider profile of differences between synaesthetes and non-synaesthetes (Bargary and Mitchell, 2008; Barnett et al., 2008a; Hanggi et al., 2011; Dovern et al., 2012). A variety of additional evidence supports this hypothesis. First, different types of synaesthesia can co-occur in individuals (Ward and Simner, 2005; Asher et al., 2009) or in different members of the same family (Barnett et al., 2008a). This led us to propose that an individual genetic mutation may either probabilistically affect wiring across the brain, giving a distinct profile in each individual, or may cause initially widespread differences in wiring, which could be resolved differently in different individuals through experience-dependent processes (Barnett et al., 2008a). Second, several studies have found differences in more general psychological characteristics between synaesthetes and controls, including mental imagery (Barnett and Newell, 2008), sensory sensitivity (Banissy et al., 2009) and significantly higher scores on positive and disorganized schizotypy (Banissy et al., 2012a). Third, we and others have detected differences in very early stages of sensory processing in visual and auditory evoked potentials (Beeli et al., 2008; Barnett et al., 2008b; Goller et al., 2009; Jancke et al., 2012), apparently independent of synaesthetic cross-activation *per se*. Finally, synaesthetes showed widespread differences in global network topology based on cortical thickness correlations (Hanggi et al., 2011) or on functional connectivity measures (Dovern et al., 2012), which were not confined to areas hypothesized to be involved in the grapheme-color synaesthetic experience itself.

To attempt to address whether the areas of structural differences are involved in the synaesthetic experience *per se*, we used the clusters of increased gray matter volume as regions of interest in an fMRI experiment. The goal was to identify regions showing differential responsiveness to letters vs. non-meaningful characters, in synaesthetes but not in controls. Our expectation was that any such differences would be caused by an increased response specifically to letters in synaesthetes, reflecting the supposed “extra activation” associated with the concurrent percept. To our surprise, the areas that did show a difference showed the opposite effect—the response to letters in synaesthetes was not just lower than to meaningless characters (and also lower than in controls), it was negative in sign. This is calculated relative to the baseline activity in the individual voxels of each cluster over the course of the experiment. It is thus not an artifact of averaging across the whole brain. As a control, we examined whole-brain responses to colors and achromatic stimuli to ensure that we could detect an expected positive BOLD response to this contrast in our experiment. We did indeed detect such a signal in the generally expected regions of ventral occipitotemporal cortex (Lueck et al., 1989; Mckeefry and Zeki, 1997; Brouwer and Heeger, 2009) (as well as a number of other regions) in both synaesthetes and controls (Table 6 and Supplementary Figure 2).

**Table 6:** **Voxelwise ANOVA main effect for condition (color/achromatic) *P* = 0.05 corrected**.

**Cluster ID**	**Volume (mm^3^)**	**Location (approximate center of mass)**	**Coordinates**			**Mean (color)**		**Mean (achrom)**	
			***X***	***Y***	***Z***	**Syn**	**Ctrl**	**Syn**	**Ctrl**
1	36,648	Left culmen/lingual gyrus	−1	−65	−6	0.129	0.098	0.332	0.298
2	2923	Left culmen/thanlamus	−2	−30	−2	0.115	0.054	0.331	0.173
3	1462	Right fusiform/middle occipital gyrus (BA19)	47	−71	−11	0.260	0.436	0.184	0.279
4	1230	Left inferior parietal lobule (BA40)	−53	−32	41	0.065	0.089	0.199	0.169
5	1046	Left inferior occipital/lingual gyrus (BA18)	−28	−89	−7	0.579	0.724	0.503	0.567
6	1003	Right superior temporal gyrus (BA39)	53	−58	24	−0.008	−0.075	0.098	0.020
7	983	Right inferior parietal lobule	52	−44	40	0.070	0.027	0.189	0.183
8	930	Right uvula/declive	25	−74	−24	0.551	0.586	0.422	0.399
9	783	Left uvula/declive	−23	−81	−21	0.637	0.543	0.504	0.393
10	744	Right middle frontal gyrus(BA8)	45	10	43	0.045	−0.069	0.144	0.076
11	626	Left superior temporal gyrus (BA39)	−53	−52	11	−0.053	−0.112	0.098	−0.001
12	588	Left medial frontal gyrus	−9	−2	49	0.126	0.177	0.241	0.252
13	585	Right cerebellar tonsil	26	−50	−40	0.127	0.035	0.230	0.135
14	487	Left middle frontal gyrus	−37	34	−3	−0.126	−0.079	0.035	0.072
15	451	Left superior temporal/pre−central gyrus (BA44)	−49	0	5	−0.130	0.026	0.070	0.158
16	423	Left brain stem	−5	−15	−26	−0.002	0.003	0.170	0.174
17	410	Right middle frontal gyrus (BA9)	35	29	31	0.023	−0.073	0.089	0.072
18	400	Right middle frontal/superior frontal gyrus (BA 10)	31	48	20	−0.072	−0.015	0.037	0.147
19	372	Right lentiform nucleus/lateral globus pallidus	13	6	2	0.028	0.044	0.180	0.205
20	372	Left superior frontal gyrus (BA10)	−23	43	26	−0.115	−0.041	0.070	0.051
21	362	Left superior frontal/ middle frontal gyrus	−34	50	15	−0.102	−0.149	0.104	0.060
22	347	Left cerebellar tonsil	−38	−56	−40	0.116	0.180	0.306	0.268
23	330	Left superior temporal/post−central gyrus (BA42)	−65	−29	19	−0.037	−0.019	0.088	0.066
24	322	Left post−central gyrus (BA 7)	−12	−50	72	−0.038	−0.024	0.020	−0.007
25	289	Left inferior parietal lobule (BA40)	−60	−46	38	0.035	−0.044	0.120	0.109

Our whole-brain analyses also revealed several areas showing a negative BOLD response to letters, but not characters, in synaesthetes, but not controls. No areas showed a similarly selective greater positive BOLD response to letters in synaesthetes. The lack of positive BOLD signals reflecting the synaesthetic experience is similar to the results of Hupe et al. (2012) and reflects the general variability in fMRI findings where such signals are not reliably found in any specific brain region (Rouw et al., 2011). The negative BOLD signals should be interpreted with caution, given that these findings were unexpected, arose in a modest sample size, and differ from most previous reports.

Nevertheless, the observation of negative BOLD signals is congruent with an early PET imaging study of auditory-color synaesthesia that also reported cortical deactivations, in addition to activations, in synaesthetes but not controls, in response to stimuli inducing synaesthesia (Paulesu et al., 1995). It would be interesting to know whether previous fMRI studies of synaesthesia did not report negative BOLD signals because they did not arise or because they were not detected due to differences in experimental design (such as focus on specific regions of interest) or analysis methods.

With the caveat that the generalizability of these findings will have to be confirmed in future studies, it is interesting to speculate on what they might mean. Negative BOLD is associated with decreased neuronal activity (Shmuel et al., 2006; Pasley et al., 2007; Wade and Rowland, 2010; Keller et al., 2013) and increased GABA concentrations (Northoff et al., 2007) and thus seems to reflect true deactivation or inhibition, as opposed to physiological artifacts such as blood stealing by nearby active areas. The induction of negative BOLD in the somatosensory cortex ipsilateral to a peripheral stimulus correlates with a reduction in perceptual sensitivity of the non-stimulated hand (Kastrup et al., 2008), reflecting neuronal hyperpolarization and increased inhibition (Devor et al., 2007). The physiological importance of such mechanisms is supported by their involvement in somatosensory habituation (Klingner et al., 2012) and implication in the enhancement of contrast between stimulated regions of visual cortex and surrounding regions with adjacent receptive fields (Wade and Rowland, 2010). In the latter case, active long-range suppressive mechanisms have been invoked to explain the emergence of negative BOLD signals.

One possible, though speculative, explanation for these observations relates to the fact that the synaesthetic percept or association is internally generated and often reported as being “in the mind's eye.” A number of studies have shown that generation of an internal sensory representation induces deactivation of regions which might compete for attention or provide conflicting information. For example, visual imagery induces negative BOLD in auditory cortex (Amedi et al., 2005), verbal memory induces deactivation across auditory and visual cortices (Azulay et al., 2009) and imagery of visual motion induces deactivation of early visual cortices (V1-3) (Kaas et al., 2010). Amedi and colleagues found a strong correlation across subjects between the deactivation of auditory cortex during visual mental imagery and their score on the vividness of visual imagery questionnaire (VVIQ). We have previously reported that synaesthetes tend to score higher on this imagery measure (Barnett and Newell, 2008). This is not to suggest that the synaesthetic percepts arise from the same processes as mental imagery *per se*—there is evidence from functional imaging that this is not the case (Rich et al., 2006; Steven et al., 2006). But it is possible that the vividness of a mental image and of a synaesthetic percept both rely on deactivation of other areas.

Such a conclusion is supported by findings from a transcranial direct current stimulation (tCDS) study. Terhune and colleagues found that synaesthetes showed enhanced cortical excitability of primary visual cortex, with a 3-fold lower phosphene detection threshold in response to activation by tCDS (Terhune et al., 2011). They tested whether this hyperexcitability of primary cortex could be either a contributing source to the generation of the synaesthetic percept, or, alternatively, a competing signal, which would interfere with the conscious perception of the synaesthetic percept. They show strong evidence that the latter is the case—stimulation or inhibition of primary visual cortical activity diminished or enhanced, respectively, the synaesthetic experience, based on both self-reports and behavioral interference measures. It thus seems plausible that the cortical deactivations we observe in response to stimuli that induce the synaesthetic experience could be an important part of that response, possibly involved in reducing the signals of competing percepts and allowing the internally generated synaesthetic percept to reach conscious awareness. Future experiments will be required to determine whether such deactivations are indeed a replicable finding and what their functional roles may be.

### Conflict of interest statement

The authors declare that the research was conducted in the absence of any commercial or financial relationships that could be construed as a potential conflict of interest.

## Supplementary material

Supplemental Figure 1 **Results for the TBSS whole brain skeletonized white matter analysis showing significant between-group increases in synaesthetes compared to non-synaesthete controls (*p* = 0.01 FDR corrected).** The significant clusters of FA difference are enhanced for visualization purposes using standard FSL TBSS visualization tools and are shown on the typical mean white matter skeleton image on the MNI 152 TI image within FSL. The slice locations for significant clusters are in shown in blue on the right of the image.

Supplemental Figure 2 **Responses to color/achromatic contrast.** Two cortical clusters, in **(A)** right fusiform/middle occipital gyrus and **(B)** left inferior occipital/lingual gyri, show increased positive BOLD responses to colored compared to achromatic stimuli, in both synaesthetes and controls.

## Abbreviations

DTI: diffusion tensor imaging
FA: fractional anisotropy
GM: gray matter
VBM: voxel based morphometry
WM: white matter.
