# Supplementary figures and images for: Combined structural and functional imaging reveals cortical deactivations in grapheme-color synaesthesia
Source: Front Psychol. 2013 Oct 30;4:755. doi: 10.3389/fpsyg.2013.00755 (PMC3812565; doi:10.3389/fpsyg.2013.00755)

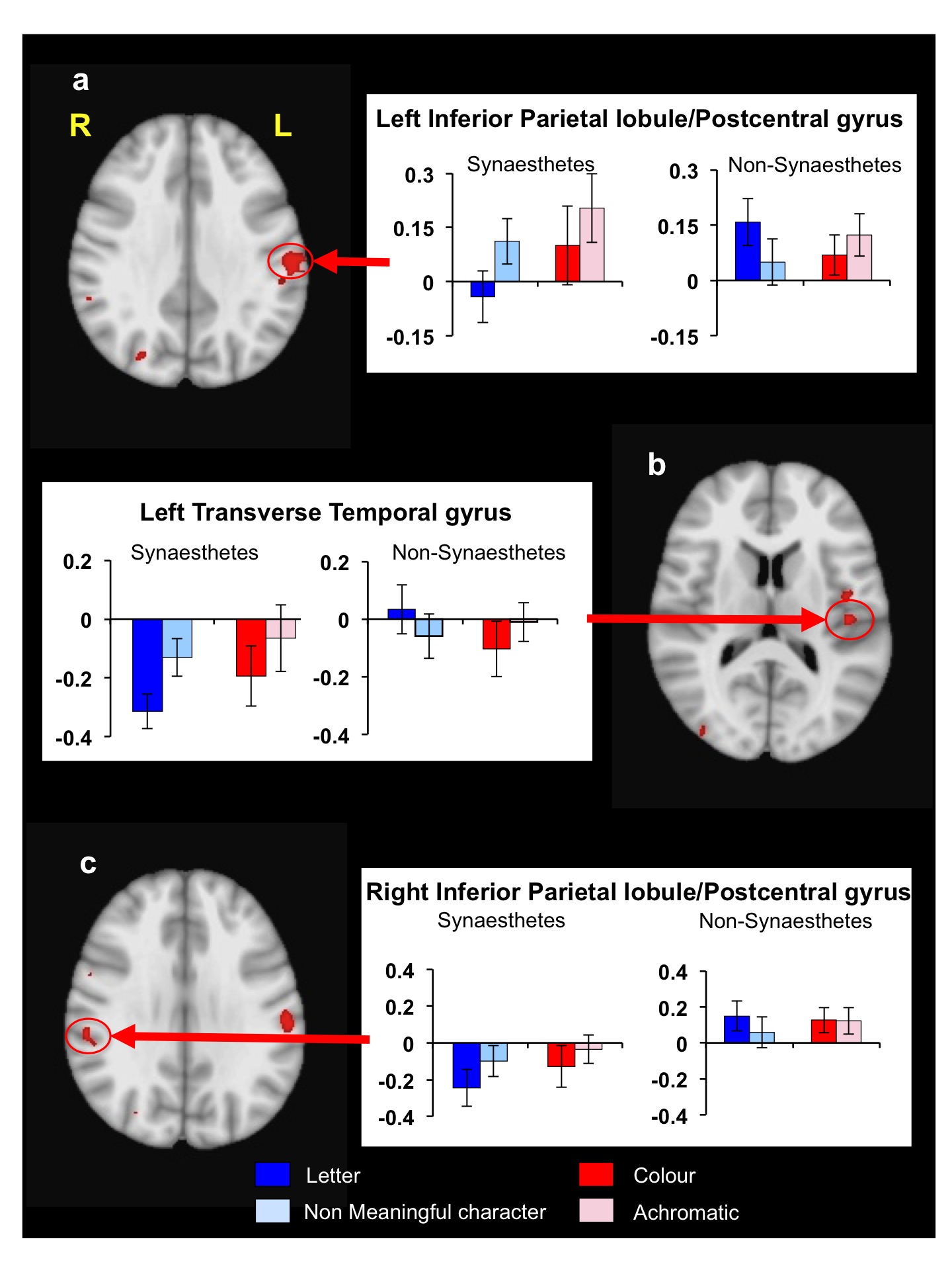

Supplement: Supplemental Figure 1 — Results for the TBSS whole brain skeletonized white matter analysis showing significant between-group increases in synaesthetes compared to non-synaesthete controls (p = 0.01 FDR corrected). The significant clusters of FA difference are enhanced for visualization purposes using standard FSL TBSS visualization tools and are shown on the typical mean white matter skeleton image on the MNI 152 TI image within FSL. The slice locations for significant clusters are in shown in blue on the right of the image. [file Presentation1.ZIP › 57046_Mitchell_S1.JPEG]

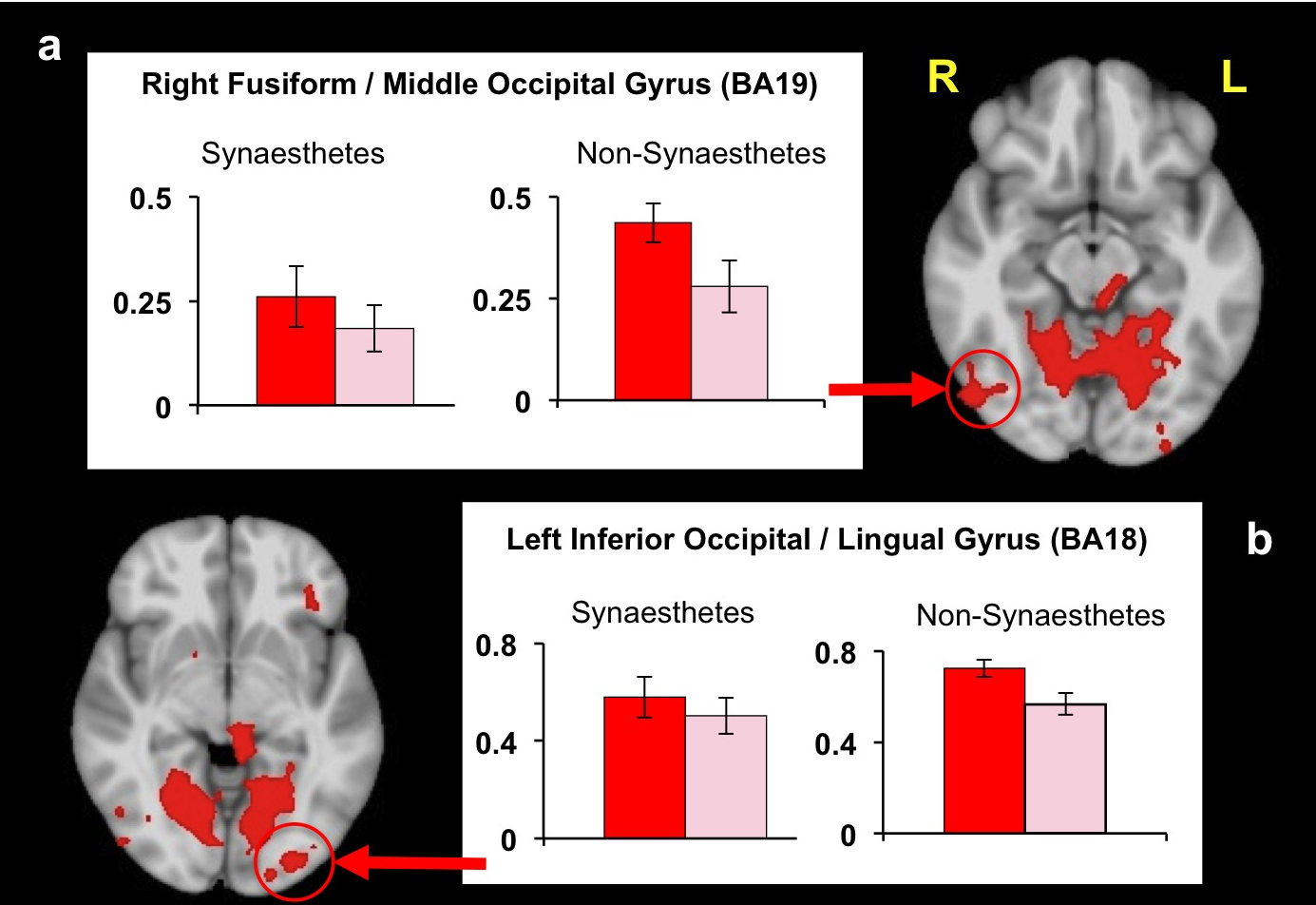

Supplement: Supplemental Figure 1 — Results for the TBSS whole brain skeletonized white matter analysis showing significant between-group increases in synaesthetes compared to non-synaesthete controls (p = 0.01 FDR corrected). The significant clusters of FA difference are enhanced for visualization purposes using standard FSL TBSS visualization tools and are shown on the typical mean white matter skeleton image on the MNI 152 TI image within FSL. The slice locations for significant clusters are in shown in blue on the right of the image. [file Presentation1.ZIP › 57046_Mitchell_S2.JPEG]
